# Downregulation of Methyltransferase-Like 14 Promotes Ovarian Cancer Cell Proliferation Through Stabilizing TROAP mRNA

**DOI:** 10.3389/fonc.2022.824258

**Published:** 2022-02-18

**Authors:** Yize Li, Hongyan Peng, Peng Jiang, Jiarui Zhang, Yongmei Zhao, Xuelian Feng, Cui Pang, Jingyi Ren, Hongmei Zhang, Wendong Bai, Wenchao Liu

**Affiliations:** ^1^ Department of Clinical Oncology, Xijing Hospital, Fourth Military Medical University, Xi’an, China; ^2^ Department of Internal Medicine, 63650 Military Hospital, Urumqi, China; ^3^ Department of Respiratory Medicine, Xinjiang Command General Hospital of Chinese People’s Liberation Army, Urumqi, China; ^4^ Department of Pathology, Tangdu Hospital, Fourth Military Medical University, Xi’an, China; ^5^ Department of Hematology, Xinjiang Command General Hospital of Chinese People’s Liberation Army, Urumqi, China; ^6^ Department of Endocrinology, Xijing Hospital, Fourth Military Medical University, Xi’an, China

**Keywords:** Ovarian cancer, Copy number variations, m^6^A RNA methylation, METTL14, TROAP

## Abstract

Altered expression levels of the proteins that regulate N6-methyladenosine (m^6^A) RNA methylation, including methyltransferase-like 14 (METTL14), are associated with cancer development. Based on our analysis of m^6^A methylation regulators using The Cancer Genome Atlas (TCGA) and Gene Expression Omnibus (GEO) datasets, we focused on the regulatory role of METTL14 in ovarian cancer. We performed bioinformatics and survival analyses with these datasets and also used METTL14-overexpressing SKOV-3 ovarian cancer cells for *in vitro* studies. Trophinin associated protein (TROAP) siRNA and treatment with or without actinomycin D was used in the cells for qRT-PCR, western blot, cDNA microarray, cell viability, colony formation, luciferase gene reporter, methylated RNA immunoprecipitation (MeRIP)-qPCR, total RNA methylation, and RNA stability assays. Additionally, ovarian cancer and normal tissue samples were analyzed by immunohistochemistry, qRT-PCR, and western blot assays. The TCGA and GEO data confirmed copy number variations (CNVs) of these m^6^A RNA methylation regulators in ovarian cancer tissues. Furthermore, reduced METTL14 expression was associated with alterations in CNVs as well as poor patient survival in ovarian cancer. Moreover, the METTL14 and m^6^A RNA methylation levels were both significantly reduced in ovarian cancer tissues than in normal tissues. Restoration of METTL14 expression suppresses ovarian cancer cell proliferation by inhibition of TROAP expression. Further *in vivo* and *in vitro* experiments confirmed that METTL14 is a negative regulator of ovarian cancer cell proliferation *via* TROAP expression and that m^6^A RNA methylation regulates TROAP mRNA stability. In conclusion, METTL14 overexpression decreased ovarian cancer proliferation by inhibition of TROAP expression *via* an m^6^A RNA methylation-dependent mechanism.

## Introduction

N6-methyladenosine (m^6^A) RNA methylation is the most prevalent RNA modification in eukaryotic cells and regulates many functions, including RNA splicing, translocation, stability, and protein translation (1). This modification is catalyzed by RNA methyltransferases known as writers, such as methyltransferase-like 14 (METTL14), and is removed by demethylases known as erasers, such as fat mass and obesity-associated protein (FTO). Additionally, m^6^A is recognized by m^6^A-binding proteins known as readers, such as IGF2BPs, which promote RNA stability and translation (2). Altogether, these proteins determine the dynamic and reversible regulation of m^6^A RNA modification (3), which can become disrupted if the expression or function of these proteins are altered, resulting in the development of human diseases and cancers (4). Aberrant expression of proteins that regulate m^6^A RNA methylation, such as METTL14, is associated with the development of various human cancers, including breast cancer (1), hepatocellular carcinoma (5), colorectal cancer (6), pancreatic cancer (7), renal clear cell carcinoma (8), bladder cancer (9), and head and neck squamous cell carcinoma (10). Furthermore, the increased expression of m^6^A RNA methylation regulators is associated with the tumorigenesis and metastasis of ovarian cancer (11) as well as gastrointestinal tract cancer (12). Therefore, a better understanding of these m^6^A writers, erasers, and readers will provide the information necessary to clarify the biological functions and potential mechanisms of m^6^A.

Ovarian cancer has the highest death rate of all gynecological malignancies due to its diagnosis at advanced stages as well as a lack of effective clinical treatments (13). Epigenetic m^6^A methylation has been reported to occur in ovarian cancer (14). However, ovarian cancer is a heterogeneous disease that is divided into many subtypes with different etiologies, pathogeneses, and prognoses. Therefore, further ovarian cancer studies are warranted to improve our knowledge on ovarian tumorigenesis and to reduce the death rate of this deadly cancer in women. Previous ovarian cancer studies have used different approaches, including analyses of aberrant gene copy number variations (CNVs) or potential molecular mechanisms (15), to better understand the development and progression of ovarian cancer. Therefore, in this study, we assessed 23 regulators of m^6^A methylation using The Cancer Genome Atlas (TCGA) and Gene Expression Omnibus (GEO) datasets to perform bioinformatics and survival analyses. Based on these analyses, we decided to focus on METTL14 in ovarian cancer tissues and cells. We transfected ovarian cancer SKOV-3 and A2780 cells with a METTL14 expression vector or Trophinin associated protein (TROAP) siRNA. The cells were then treated with or without actinomycin D for various assays to understand the *in vivo* and *in vitro* regulatory roles of METTL14 in ovarian cancer as well as the underlying molecular events. The results of our study provide important insights into the m^6^A RNA modification by METTL14 in the regulation of ovarian cancer.

## Materials and Methods

### Data Resources and Bioinformatic Analysis

The transcriptomic RNA-seq data of 295 ovarian cancer patients was downloaded from cBioPortal for cancer geonomics (https://www.cbioportal.org/study/summary?id=ov_tcga_pan_can_atlas_2018), combined TCGA pancancer atlas studies and followed with clinical data in website. The data on the CNVs of 23 m^6^A RNA methylation regulators in ovarian cancer was collected, according to previous studies (16). The datasets on the mRNA expression levels of m^6^A RNA methylation regulators as well as METTL14 were downloaded from the GEO database (GSE14407 and GSE119168, respectively). Next, the data was analyzed using the GEO database-supported ClueGO+CluePedia tool in Cytoscape for the cluster-envisioning characterization of the biological processes (i.e., dysregulation or downregulation) of these m^6^A RNA methylation genes in ovarian cancer and the functional categories of the gene ontology (GO) and the Kyoto Encyclopedia of Genes and Genomes (KEGG) databases using the DAVID bioinformatics resource website (https://david.ncifcrf.gov/). The GO terms of the GEO data were assessed using the PANTHER website (http://www.pantherdb.org/) for functional gene categories and associations between genes and the corresponding GO classifications. The R visualization package GOPlot was then used to obtain a better visualization of the relationships between the genes and the selected functional categories. Next, the correlation between METTL14 and TROAP expression levels was obtained from the GEO dataset (GSE28724), and the datasets and tools provided by KMplot were used to analyze the survival of patients with high and low METTL14 levels (http://kmplot.com/analysis/index). In addition, the Human Protein Atlas database was used to verify the translation of TROAP (http://www.proteinatlas.org/), and the m^6^A RNA methylation sites were predicted by using the m^6^AVar prediction website (http://m6avar.renlab.org/index.html).

### Tissue Samples and Cell Culture

Normal (n = 20) and cancerous (n = 20) ovarian tissue samples were collected at the Xijing Hospital (Xi’an, China) between May and November 2019. All patients were histologically diagnosed with epithelial ovarian cancer. The tissue specimens were obtained immediately after resection and then divided into three portions for RNA isolation, protein extraction, and storage in liquid nitrogen for further analyses. The experimental protocol was approved by the Ethics Committee of Xijing Hospital, The Fourth Military Medical University. The ovarian cancer SKOV-3 and A2780 cell lines was obtained from the Shanghai Institutes of Biological Sciences (Chinese academy of sciences, Shanghai, China) and maintained in Roswell Park Memorial Institute 1640 Medium supplemented with 10% heat-inactivated fetal bovine serum, penicillin (10 U/mL), and streptomycin (50 µg/mL) at 37°C in a 5% CO_2_ atmosphere.

### Plasmid Construction, Lentivirus Production, and Cell Transduction

The pMD-18T plasmid carrying METTL14 cDNA was constructed by PCR amplification with the following primers: 5′-ATGGATAGCCGCTTGCAGGAGATCCGGG-3′ and 5′-TTATCGAGGTGGAAAGCCACCTCTGTG-3′. The resulting product was then subcloned into pLenti-6.3 (Invitrogen, Carlsbad, CA, USA) for lentiviral production. The lentivirus was packaged and used to transduce the SKOV-3 cells, according to the standard protocols recommended by the manufacturer. To generate the ovarian cancer cell line with stable METTL14 expression, SKOV-3 cells were grown and transduced with the lentivirus carrying the METTL14 expression vector, or only the vector as a control, and then treated with puromycin (1.5 ug/mL) for 2 weeks for cell selection. The METTL14 cDNA was cloned into the pC3 plasmid (Invitrogen) for transfections.

### Small-Interfering RNA and Cell Transfection

TROAP siRNA (si-TROAP) and Wilms tumor-associated protein (WTAP) siRNA (si-WTAP) was obtained from GenePharma Inc. (Shanghai, China). Briefly, SKOV-3 cells were cultured and transiently transfected with si-TROAP or negative control oligonucleotides at a final concentration of 100 nM using Lipofectamine 2000 (Invitrogen) for 24–48 h. The cells were then collected for quantitative reverse transcription (qRT)-PCR and western blot analyses (17). The siRNA sequences were as follows: (TROAP) 5′-GTAGGATTGAGCCTGAGAT-3′, (WTAP) 5′-GGAGGUAGTGGUUACGUAAAU-3′, and (negative control) 5′-UUCUCCGAACGUGUAACGUTT-3′ (18).

### Plasmid Construction and Luciferase Reporter Assay

The pMD-18T vectors carrying either the TROAP or METTL14 3′-untranslated region (UTR) were constructed by PCR amplification. The 5′-CCCTGCCCCTGTGGCCCAGCCCTTG-3′ and 5′-CTGAGTGTTTTAACAGTCCAG-3′ primer sequences were used to amplify the 3′-UTR of the 575-bp TROAP fragment containing the m^6^A methylation site. The product was then subcloned into the pMD-18T vector (Takara bio, Shiga, Japan). The vector was amplified and sequenced before use. Similarly, the METTL14 3′-UTR fragments were also cloned into the pMD-18T vector. These pMD-18T vectors were used to release the inserts, which were then subcloned into pGL3 vectors (Promega, Madison, WI, USA). Additionally, the wild-type METTL14 3′-UTR m^6^A methylation site (GGA; pGL3-WT-UTR) was mutated to CGT (pGL3-Mut-UTR). After amplification and DNA sequence confirmation, these vectors were used to transfect SKOV-3 cells. Specifically, SKOV-3 cells were seeded into 48-well plates as duplicates, grown for 24 h, and then co-transfected with 1 µg pGL3-WT-UTR (TROAP 3′-UTR containing the m^6^A methylation site) or pGL3-Mut-UTR as well as 1 µg renilla luciferase vector pRL-TK for 48 h. The total protein lysates were obtained using a kit from Promega and used for the luciferase assay, according to the dual-luciferase reporter assay method (19). Firefly luciferase activity was used as a normalization control for each sample.

### qRT-PCR

Total cellular RNA was isolated from cells and tissues using Trizol reagent (Invitrogen), and cDNA was obtained by reverse transcription using SuperScript II reverse transcriptase (Invitrogen), according to the manufacturer’s recommended protocol (20). Next, the relative mRNA expression levels of METTL14, TROAP, and GAPDH (as a normalization control) were assessed using SYBR Premix Ex Taq II (Takara, Dalian, China). The primer sequences were as follows: (METTL14) 5′-GAACACAGAGCTTAAATCCCCA-3′ and 5′-TGTCAGCTAAACCTACATCCCTG-3′, (TROAP) 5′-CCTCCGGGGTGTATCTCCTAC-3′ and 5′-ACGGCGCACGATGTAACAG-3′, (WTAP) 5′-CTTCCCAAGAAGGTTCGATTGA-3′ and 5′-TCAGACTCTCTTAGGCCAGTTAC-3′, (GAPDH) 5′-GGAGCGAGATCCCTCCAAAAT-3′ and 5′-GGCTGTTGTCATACTTCTCATGG-3′.

### Western Blot Analysis

Western blot analysis was used to determine changes in protein levels, according to a previous study (14). The primary antibodies used were rabbit polyclonal anti-TROAP (Cat. #HPA044102; Sigma-Aldrich, St. Louis, MO, USA), anti-METTL14 (Cat. #ab220031; Abcam, Cambridge, MA, USA), anti-cyclin D1 (Cat. #ab16663; Abcam), anti-survivin (Cat. # ab76424; Abcam), anti-p-AKT (Cat. #ab38449; Abcam), anti-WTAP (Cat. #ab195380; Abcam), and polyclonal rabbit anti-TROAP (Cat. #13634-1-AP; Proteintech, Rosemont, IL 60018, USA). The Odyssey infrared imaging system (LI-COR Biosciences, Lincoln, NE, USA) was used for protein imaging.

### Cell Viability and Colony Formation Assays

Cell viability was measured using the CellTiter 96 Aqueous One Solution Cell Proliferation Assay kit (Promega). Briefly, tumor cells (5 × 10^3^) were seeded into the individual wells of a 96-well plate and cultured for 24 h. The cells were then subjected to the assay, according to the manufacturer’s protocol. Furthermore, the colony formation capacities of SKOV-3 and A2780 cells after transfection were assayed, as previously described (21).

### Methylated RNA Immunoprecipitation-qPCR

MeRIP-qPCR was performed to quantify the m^6^A-modified levels of TROAP. Specifically, total cellular RNA was isolated from SKOV-3 cells using Trizol regent. The RNA samples (100 μg for each reaction) were incubated with 3 μg anti-m^6^A (Cat. #208577; Abcam, Cambridge, MA, USA) or anti-IgG (Cat. #172730; Abcam) that were conjugated with A/G magnetic beads in an immunoprecipitation buffer containing RNase inhibitor and protease inhibitors. Next, RNA from the mixtures was collected through centrifugation, cleaned using a phenol-chloroform solution, and eluted with elution buffer. The RNA samples (10 ng for each reaction) were then subjected to reverse transcription using Superscript III random hexamers (Invitrogen, Shanghai, China), and the cDNA was further analyzed by qPCR. The TROAP primer sequences that were used for MeRIP-qPCR were 5′-TTGCGGCGTCTCACCGTTCAACCT-3′ and 5′-GCCTCCATTAAGAGGGACACACTGG-3′. TROAP mRNA levels were quantified using the 2^-ΔΔCt^ associated sample addition method for fold enrichment.

### RNA Stability Assay

SKOV-3 cells with or without METTL14 overexpression were treated with actinomycin D at a final concentration of 5 ug/mL for 0.5, 1, 2, or 4 h. The cells were then collected for total RNA isolation for qRT-PCR analysis. The turnover rate and half-life of mRNAs were then determined according to a previous study (22).

### Tissue Microarray and Immunohistochemistry Assays

A TMA containing 12 healthy endometrial tissues and 58 ovarian cancer tissues was purchased from Servicebio (Wuhan, China; Cat. #OC-1601). Immunohistochemistry was used to assess the expression of METTL14. Briefly, the TMA sections were deparaffinized in xylene, rehydrated in a series of ethanol solutions, and then subjected to antigen retrieval in 10 mM sodium citrate/0.05% Tween 20 (pH 6). The TMA sections were then incubated with anti-METTL14 antibody (Cat. #ab220030; Abcam), and the vectastain elite ABC HRP and DAB substrate kits were used for immunohistochemical detection (Vector laboratories, CA, US), according to the manufacturer’s recommended protocols. Hematoxylin was used to stain the cell nuclei. Next, the immunostained TMA sections were reviewed, photographed, and quantified using the ImageJ plugin IHC profiler to determine the grade of epithelial component staining (0, no staining; 1, low positive staining; 2, positive staining; 3, high positive staining).

### Immunohistochemistry and Fluorescence *In Situ* Hybridization

IHC performed with ovarian cancer tissue microarray and animal tissue specimens. For IHC, the samples were incubated with primary antibody, followed by incubation with HRP-conjugated secondary antibody. METTL14 FISH was performed to analyze the copy numbers of the METTL14 gene in ovarian cancer and normal tissue samples. Briefly, tissue sections were deparaffinized in xylene, rehydrated in a series of ethanol solutions, and then subjected to FISH with the METTL14 FISH probe (1–2.5 nM) in a buffer containing 2× saline-sodium citrate, 25% formamide, 10% dextran sulfate, and 1 mg/mL yeast in a humidified chamber for 3–4 h at 42°C, according to the manufacturer’s recommended protocol. The specific FISH signal was detected as brown punctate dots. Images were captured using a Nikon 90i microscope-equipped orca ER camera (Hamamatsu Photonics, Hamamatsu, Japan) with a 60×, 1.4 N.A. VC objective lens and were quantified using the Volocity visualization software (Perkin elmer, Waltham, MA, USA).

### Animal Experiments

Six-week-old BALB/c female nude mice were housed in a pathogen-free animal facility under normal conditions. All animal experimental procedures were approved by the Animal Care Committee of the Fourth Military Medical University. SKOV-3 cells (1 ×10^6^) stable transfected with METTL14 or control lentivirus, were injected subcutaneously into the right flank of BALB/c nude mice. Tumor growth was monitored every 5 days for a total period of 25-40 days.

### Statistical Analysis

Continuous variable data were summarized as the mean ± standard error of the mean from at least three independent experiments and analyzed using Student’s *t*-tests. Associations of the changes in the expression levels of m^6^A RNA methylation regulators with the clinicopathological or distinguishing molecular features of cancer patients were assessed using the χ^2^ or Fisher’s exact tests. One-way analysis of variance (ANOVA) or Student’s *t*-tests were performed to analyze individuals who were clustered by clinical or molecular-pathological criteria in relation to the ovarian cancer signature incidence rates for specific clinical conditions. Kaplan-Meier curves and the log-rank test were used to analyze the significance of m^6^A RNA methylation, CNVs, and mutations in association with overall patient survival. Survival information was acquired by patient follow-up, and the information from the last follow-up was used for these analyses. All statistical analyses were performed using Prism version 7.0 or R version 3.4.1 (https://www.r-project.org/) of GraphPad (San Diego, CA, USA). A *P*-value ≤ 0.05 was considered to be statistically significant.

## Results

### METTL14 Was Downregulated in Ovarian Cancer and Predicted Poor Patient Survival

In this study, we assessed the TCGA datasets for the mutations of each individual m^6^A RNA methylation regulator in ovarian cancer, including AlkB homolog 5 (ALKBH5), E74 like ETS transcription factor 3 (ELF3), FMRP translational regulator 1 (FMR1), FTO, Heterogeneous nuclear ribonucleoprotein (HNRNP)A2/B1, HNRNPC, Insulin like growth factor 2 mRNA binding protein (IGF2BP) 1, IGF2BP2, IGF2BP3, Vir like m6A methyltransferase associated (Also known as KIAA1429), Leucine rich pentatricopeptide repeat containing (LRPPRC), METTL3, METTL14, RNA binding motif protein (RBM) 15, RBM15B, RBMX, WTAP, YTH N6-methyladenosine RNA-binding protein (YTHDF)1, YTHDF2, YTHDF3, YTH domain-containing (YTHDC)1, YTHDC2 and Zinc finger CCCH-type containing 13 (ZC3H13). We analyzed the CNVs of these m^6^A RNA methylation regulators in 295 ovarian cancer patients who completed the recommended diagnostic next-generation sequencing-based gene panel test.

**Figure 1:**
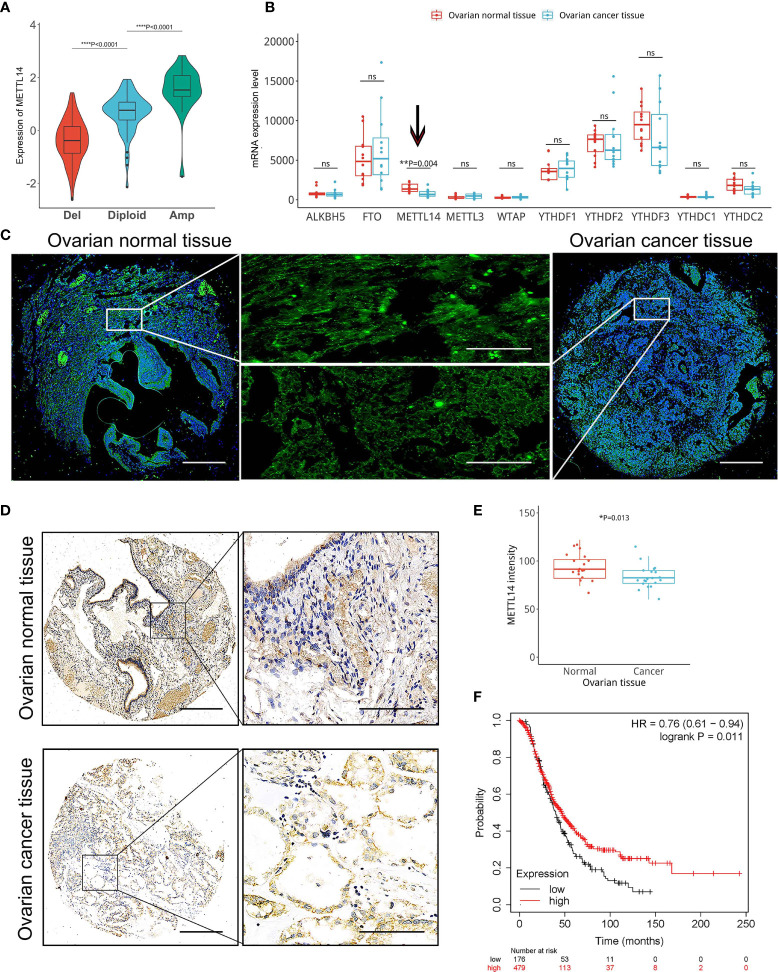
Correlation among reduced METTL14 copy number variations (CNVs) and decreased METTL14 mRNA and protein expression levels in ovarian cancer tissues. **(A)** The TCGA dataset and available tools were used for the correlation analysis of the METTL14 copy number (deletion, diploid and amplification) and mRNA expression levels (****P < 0.0001 by ANOVA). **(B)** Expression of METTL14 in ovarian cancer tissues vs. normal tissues in the GEO database (GSE14407 with METTL14, probe ID 235552_at, **P = 0.004). ns, not significant. **(C)** FISH images of METTL14 copy numbers in normal and ovarian cancer tissues. Magnification: 50 × (left and right) and 400 × (middle). **(D)** Immunohistochemistry of METTL14 expression in the tissue microarray of normal and ovarian cancer tissues. Magnification: 50 × (left) and 400 × (right). **(E)** Summarized data of **(D)** (*P=0.013). Graphical data represented as the mean ± SD. **(F)** Kaplan-Meier overall survival curves stratified by METTL14 expression levels in 655 ovarian cancer patients. The mean METTL14 mRNA value was used to assign patients to two subgroups (ID 236060_at). The log-rank test was used to calculate the differences between the two groups (*P = 0.011).

We found a significant association between the CNVs of the 23 m^6^A RNA methylation regulators, including METTL14, and their mRNA expression levels (**Figure 1A** and **Supplemental Figure S1**). Our data demonstrated that increased copy numbers coincided with elevated mRNA expression levels, whereas decreased copy numbers coincided with reduced mRNA expression levels. To verify this relationship between CNVs and mRNA levels, we evaluated the expression of 10 key m^6^A RNA methylation regulators in normal and cancerous ovarian tissues. Because a lot of shallow deletion was shown in METTL14, we hypothesized that the reduction in its copy number would result in decreased METTL14 mRNA expression levels. Among the 10 key m^6^A RNA methylation regulators, the GEO data only showed a reduction in the METTL14 mRNA levels in the ovarian cancer tissues (**Figure 1B**), which was associated with a lower METTL14 copy number. Through further analysis of the comparative genomic hybridization data using high-resolution microarrays, we found lower METTL14 copy numbers in ovarian cancer tissues than in normal ovarian tissues (**Figure 1C**). Immunohistochemical analysis of the METTL14 expression pattern using the ovarian cancer tissue array was consistent with the online database data, showing reduced METTL14 expression in ovarian cancer tissues (**Figures 1D, E** **)**. Moreover, Kaplan-Meier analysis showed that patients with reduced METTL14 expression had a lower overall survival (**Figure 1F**).

### Downregulated METTL14 Induced Aberrant m^6^A Modification and May Be Correlated With Tumor Cell Proliferation

**Figure 2:**
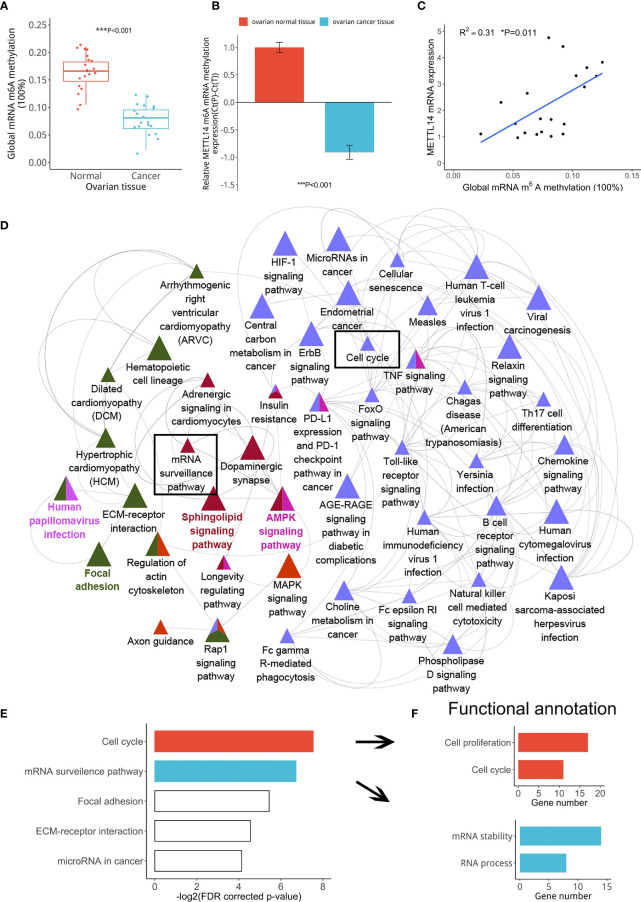
Correlation among reduced METTL14 expression and ovarian cancer cell proliferation. **(A)** Global mRNA m^6^A methylation levels from ovarian cancer or normal tissues (***P < 0.001). **(B)** MeRIP-seq data showing the METTL14 mRNA levels in the GEO database (GSE119168) with the RNA probe (ID: 118685469) (***P < 0.001). **(C)** Global mRNA m^6^A methylation levels of ovarian cancer tissues were found to be associated with METTL14 mRNA levels (*P = 0.011). Graphical data represented as the mean ± SD. **(D)** Pathway enrichment analysis using the ClueGO and CluePedia plugins found in the Cytoscape software. Cluster analysis showing the most enriched pathways according to the m^6^A RNA methylation expression levels using the GSE119168 data. **(E)** Classification of the gene categories from cluster analysis mainly included the cell cycle, mRNA surveillance pathway, and related signaling pathways. **(F)** KEGG gene functional annotation analysis using DAVID classified the gene categories and signaling pathways from two biological processes.

The m^6^A modification is mainly catalyzed by m^6^A methyltransferases and demethylases (23). Therefore, we speculated that abnormal m^6^A modification could be induced by an imbalance between key m^6^A methyltransferases or demethylases. Thus, we assessed the global m^6^A levels and found that the m^6^A levels were significantly lower in ovarian cancer tissues than in normal ovarian tissues (**Figure 2A**). Because METTL14 is an m^6^A RNA methylation writer, its reduced expression led to a decrease in its writer methyltransferase function. Therefore, we first assessed the METTL14 m^6^A methyltransferase levels using the GEO database (GSE119168) and found its levels to be significantly decreased in ovarian cancer tissues (**Figure 2B**). Our linear data analysis also showed a positive correlation between METTL14 mRNA levels and the global m^6^A levels in ovarian tissues (**Figure 2C**). These results indicated that downregulated METTL14 levels resulted in the low m^6^A RNA methylation levels in the ovarian cancer tissues. Further GEO database (GSE119168) analysis showed that most genes with low levels of m^6^A RNA methylation in ovarian cancer tissues were enriched in cell cycle, cell proliferation progression, mRNA stability, mRNA process and other pathways (**Figures 2D–F**). These results suggest that METTL14 may function as a negative regulator of tumor cell proliferation *via* its m^6^A methyltransferase function.

### METTL14 Overexpression Inhibited the Proliferation of Ovarian Cancer Cells *In Vitro*

**Figure 3:**
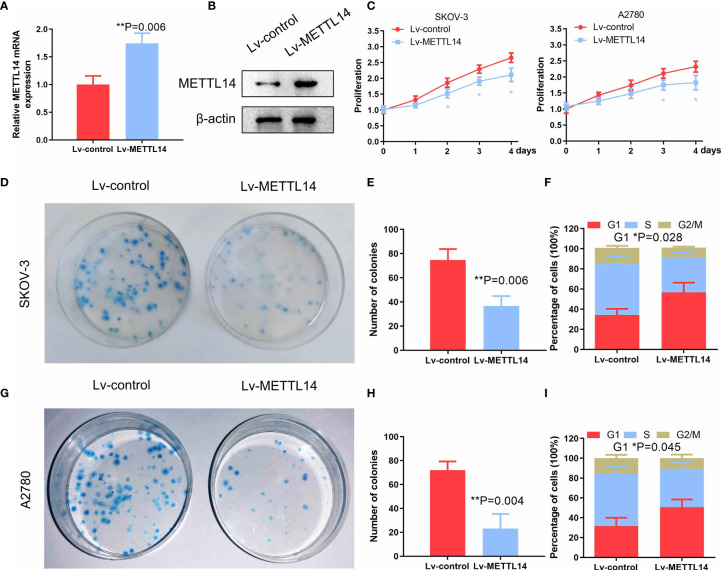
Inhibitory effects of METTL14 overexpression by lentiviral transduction on ovarian cancer cell proliferation and colony formation. **(A)** METTL14 mRNA levels of ovarian cancer cells after transduction with Lv-control and Lv-METTL14 (**P = 0.006). **(B)** Western blot of METTL14 protein levels of ovarian cancer cells after transduction with Lv-control and Lv-METTL14. **(C)** Lv-control and Lv-METTL14 transduced SKOV-3 and A2780 cells were seeded into 96-well plates, cultured for 4 days, and then subjected to an MTS cell proliferation assay (*P < 0.05). **(D, E)** Lv-control and Lv-METTL14 transduced SKOV-3 cells were seeded into 3.5-cm plates, cultured for 14 days, and then subjected to a colony formation assay (**P = 0.006). **(F)** Lv-control and Lv-METTL14 transduced SKOV-3 cells were cultured in serum-free medium for 24 h, cultured in complete growth medium for an additional 16 h, and then collected for flow cytometry to determine cell cycle distribution (*P = 0.028). **(G–I)** Lv-control and Lv-METTL14 transduced A2780 cells, subjected to a colony formation assay (**P = 0.004) and cell cycle distribution. Bar graph displaying the percentages of cells within the G1, S, and G2/M phases (*P = 0.045). Graphical data represented as the mean ± SD.

We transduced SKOV-3 cells with the lentivirus containing the METTL14 expression vector to evaluate the effect of METTL14 overexpression on the regulation of ovarian cancer cell proliferation *in vitro*. We confirmed that the METTL14 expression levels were increased after lentiviral transduction by qPCR and western blot (**Figures 3A, B** **)**. We further demonstrated that the cell viability (**Figure 3C**) and colony formation capacity of the tumor cells were reduced with METTL14 overexpression (**Figures 3D, E** **)**. Further distribution analysis by flow cytometry showed that SKOV-3 cells were arrested at the G1 phase of the cell cycle in response to METTL14 overexpression. In addition, the percentages of cells at the S and G2/M phases were decreased accordingly (**Figure 3F**). The similar results of colony formation assay and flow cytometry in A2780 cells (**Figures 3G–I**) further suggest that METTL14 may function as a negative regulator of ovarian cancer cell proliferation.

### METTL14 Downregulated TROAP Expression in Ovarian Cancer Cells and Tissues

**Figure 4:**
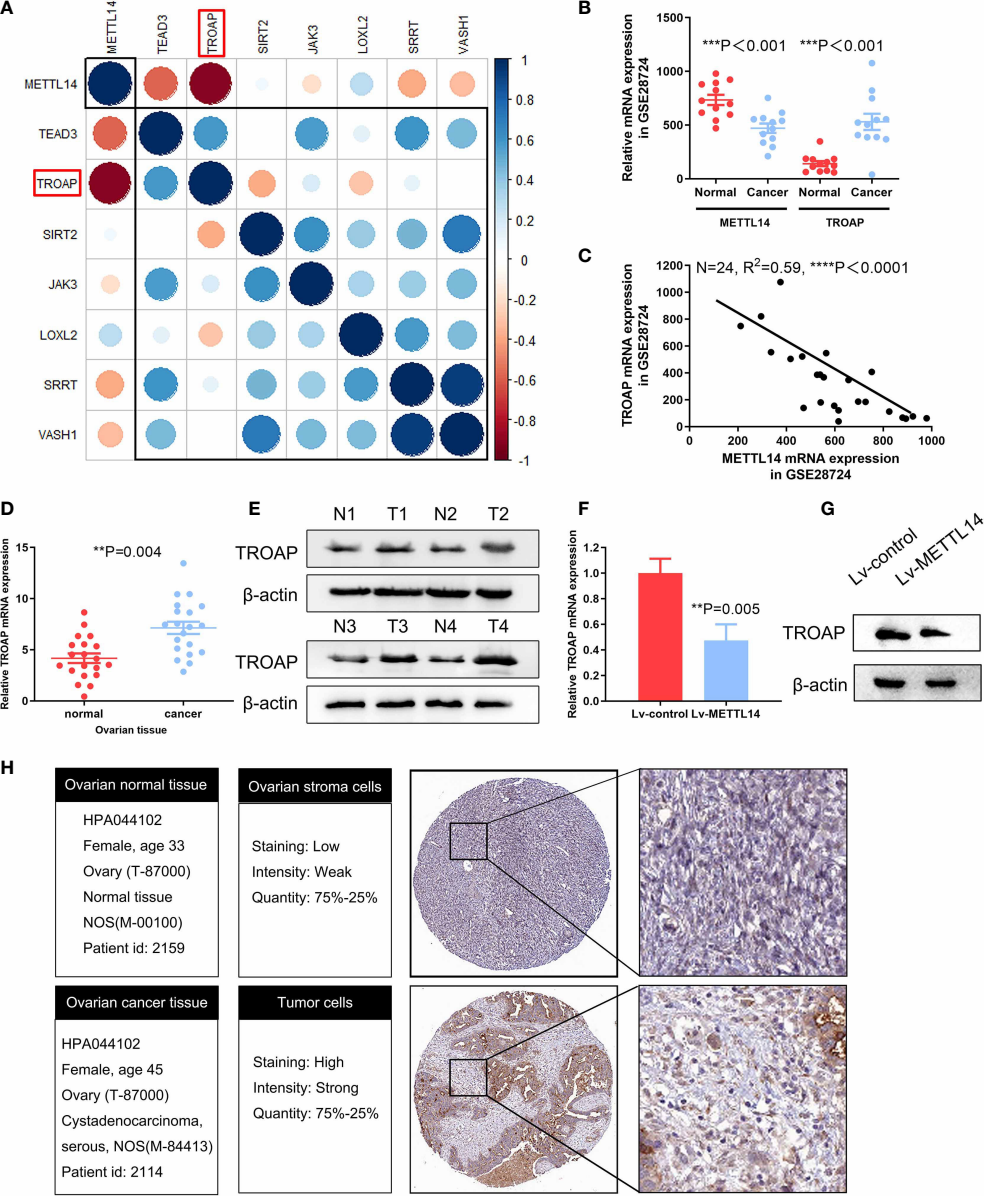
Confirmation of TROAP as a downstream target of METTL14. **(A)** Correlation matrix of candidate genes for each biological process phenotype stratified by expression levels of cell proliferation-related genes, which were measured by qRT-PCR. Blue indicates a positive correlation, and red indicates a negative correlation. In addition, brightness is proportional to correlation strength. TROAP is indicated in the red box. **(B)** Graph displaying the negative correlation between TROAP (probe ID: 202265_at) and METTL14 (probe ID: 241689_at) expression in 34 types of ovarian cancer cell lines from the GEO dataset (GSE28724) (***P < 0.001). **(C)** Expression levels of TROAP are negatively correlated with METTL14 in human ovarian cancer lines GEO dataset (GSE28724) (****P < 0.0001). **(D, E)** Analysis of TROAP at the mRNA level **(D)** and protein level **(E)** in normal and cancer ovarian tissue. **(F)** TROAP mRNA expression levels in METTL14-overexpressing SKOV-3 cells (**P = 0.005). Graphical data represented as the mean ± SD. **(G)** Western blot of TROAP protein expression levels. **(H)** Comparison of relative TROAP expression between ovarian cancer and normal tissues in The Human Protein Atlas database.

Since METTL14 had a profound impact on ovarian cancer cell proliferation, we reasoned that the specific genes regulated by METTL14 should predominantly function in this process. We firstly analyzed the inversely correlated genes with METTL14 from the GEO database (GSE14407) (Supplementary data1). According to cytoscape gene ontology analysis, these genes were involved in several biological processes, including cell proliferation (**Supplementary Figure S2**). From these data, we selected candidate genes involved in the regulation of cell proliferation for further analysis by qRT-PCR and then constructed a gene-gene correlation matrix using the expression patterns of these genes. Interestingly, we identified a negative correlation between the expression of METTL14 and the cell proliferative gene TROAP (**Figure 4A**). We postulated that the METTL14-TROAP axis may be responsible for the regulation of ovarian cancer cell proliferation. mRNA expression profiles in GEO database (GSE28724) indicated that suppressed METTL14 expression inversely correlated with TROAP (**Figures 4B, C** **)**. Thus, we performed qRT-PCR and wester blotting and observed a dramatic increased TROAP expression in ovarian cancer tissues (**Figures 4D, E** **)**. Furthermore, ectopic expression of METTL14 significantly reduced TROAP expression in SKOV-3 cells compared with the control cells (**Figures 4F, G** **)**. To investigate whether these changes in protein expression levels occur in tumor tissues, TROAP immunohistochemical staining of normal and ovarian cancer tissues were validated within Human Protein Atlas project (**Figure 4H**). Altogether, these data suggest that METTL14 functions as a negative regulator of TROAP in ovarian cancer cells and tissues.

### METTL14 Inhibited Ovarian Cancer Cell Proliferation by Targeting TROAP

**Figure 5:**
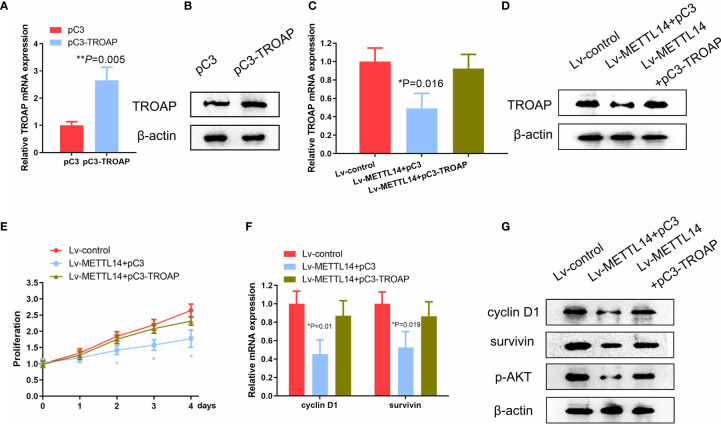
METTL14 suppresses the proliferation of ovarian cancer cell *via* TROAP. **(A, B)** TROAP mRNA and protein expression transduced with pC3 or pC3-TROAP (**P=0.005). **(C, D)** Analyses of TROAP mRNA and protein expression levels in METTL14-overexpressing SKOV-3 cells transduced with pC3 or pC3-TROAP (*P = 0.016). **(E)** MTS assay of METTL14-overexpressing SKOV-3 cells transduced with pC3 or pC3-TROAP. Values at the indicated timepoints are provided as the mean absorbance with a standard deviation of 6 wells (*P < 0.05). **(F)** Cyclin D1 and survivin mRNA levels in METTL14-overexpressing SKOV-3 cells with or without TROAP ectopic expression (*P = 0.01, *P = 0.019). Graphical data represented as the mean ± SD. **(G)** Analysis of expression levels of cyclin D1, survivin, and p-AKT protein by using western blot.

Because we found TROAP to be a functional downstream target of METTL14 in SKOV-3 cells, we next evaluated if the reintroduction of TROAP into the METTL14-overexpressing cells could reverse the inhibitory effects of METTL14 on cell proliferation. To test this hypothesis, firstly, we stably transfected SKOV-3 cells with TROAP plasmid, increasing the mRNA and protein expression of TROAP compared with control cells (**Figures 5A, B** **)**. We then transfected exogenous TROAP into the METTL14-overexpressing ovarian cancer cells. As shown in **Figures 5C, D**, the expression of TROAP in the METTL14-overexpressing cells significantly increased after TROAP transfection. Although METTL14 overexpression decreased the proliferation of SKOV-3 cells (**Figure 5E**), the overexpression of TROAP reversed this inhibitory effect on cell proliferation in the SKOV-3 and A2780 cells (**Figure 5E** and **Supplementary Figure S3**).

We next evaluated the expression of TROAP target genes, such as cyclin D1, survivin, and p-AKT, because their downregulation may also be involved in the inhibition of ovarian cancer cell proliferation due to METTL14 (24–26). As expected, the exogenous expression of METTL14 downregulated the expression levels of cyclin D1, survivin, and p-AKT. However, transfection with TROAP increased both the mRNA and protein levels of these genes compared with control cells (**Figures 5F, G** **)**. Taken together, these data suggest that METTL14 may function as a negative regulator of ovarian cancer cell proliferation *via* inhibition of TROAP and its downstream targets.

### METTL14 Decreased TROAP Stability Through m^6^A Methylation

**Figure 6:**
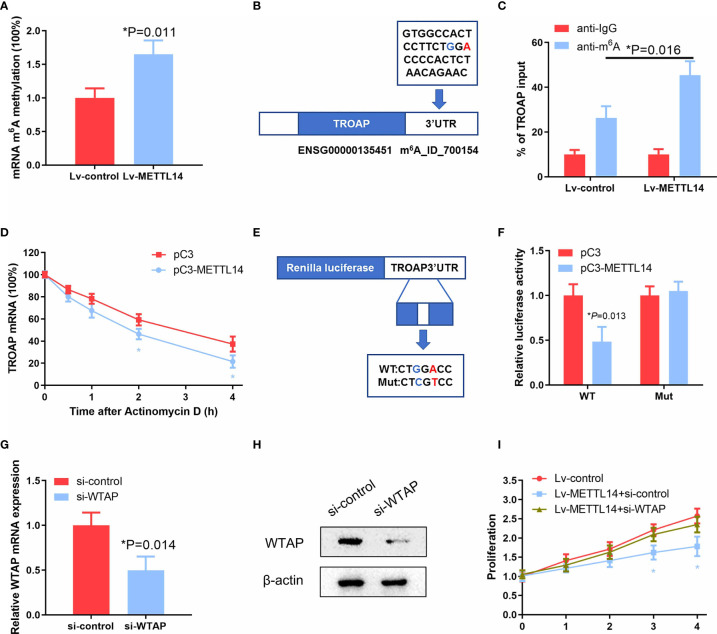
METTL14 destabilize TROAP mRNA through the m^6^A mRNA methylation. **(A)** Detection of mRNA m^6^A methylation in SKOV-3 cells transfected with the METTL14 expression plasmid (*P = 0.011). **(B)** Predicted m^6^A RNA methylation site using the m^6^AVar prediction website. **(C)** Enrichment of m^6^A-modified TROAP in METTL14-overexpressing SKOV-3 cells. Input percentage is shown (*P = 0.016). **(D)** Changes in the m^6^A-modified TROAP levels with METTL14 overexpression. SKOV-3 cells were transfected with pC3 or pC3-METTL14 plasmids for 36 h. The stabilization of mRNA was initiated by treatment with an RNA polymerase II inhibitor [actinomycin D (5μg/ml)]. Cells were collected at the indicated timepoints. At 0 h, the expression rates were normalized to that of GAPDH mRNA. Data are shown as the average of three or more different studies (*P < 0.05). **(E)** Representative cloning scheme for the wild-type and mutant TROAP 3′ UTR renilla luciferase vectors. **(F)** Co-transfection of these luciferase constructs with pC3 or pC3-METTL14 expression vectors into SKOV-3 cells. Data are shown as the average of three or more independent experiments (*P = 0.013). **(G, H)** Analyses of WTAP expression levels by qRT-PCR and western blot in SKOV-3 cells transfected with si-control or si-WTAP (*P = 0.014). **(I)** Cell proliferation analysis of stable METTL14-overexpressing SKOV-3 cells transfected with si-control or si-WTAP (*P < 0.05). Graphical data represented as the mean ± SD.

To further verify whether METTL14-induced downregulation of TROAP expression is dependent on m^6^A mRNA methylation in ovarian cancer tissues and cells, we assessed the m^6^A methylation rate in ovarian cancer cells after transfection with the METTL14 expression vector. Our results showed that METTL14 overexpression significantly elevated the rate of m^6^A RNA methylation in ovarian cancer SKOV-3 cells (**Figure 6A**). To determine the possible effects of the elevated m^6^A methylation on TROAP expression, we determined the m^6^A methylation site of TROAP mRNA using the m^6^AVar online tool, which predicts the sequence-based m^6^A alteration sites. We found an GGA m^6^A sequence motif in the TROAP 3′-UTR proximal to the stop codon (**Figure 6B**). In addition, METTL14 overexpression induced the accumulation of m^6^A methylation in this region of the TROAP mRNA (**Figure 6C**). Next, the half-life of TROAP mRNA was measured in SKOV-3 cells after transfection with the METTL14 expression vector and treatment with actinomycin D. We found that METTL14 had a strong decay effect on the TROAP mRNA (**Figure 6D**). To further verify that METTL14 functions at the m^6^A methylation site of the TROAP 3′-UTR to reduce the expression of its mRNA, we constructed luciferase reporter vectors carrying either the wild-type or mutated TROAP 3′-UTR (**Figure 6E**) and found that METTL14 overexpression resulted in the decreased luciferase activity of the wild-type TROAP 3′-UTR, whereas mutation of this m^6^A methylation site from A to T almost completely blocked the inhibitory effect of METTL14 overexpression (**Figure 6F**).

Recently, METTL3 and METTL14 have been identified to interact with WTAP, which acts as a scaffold by binding to the methyltransferases to form a complex that mediates m^6^A RNA methylation (18). Because the loss of WTAP significantly blocks the m^6^A RNA modification process, we knocked down WTAP with siRNA in METTL14-overexpressing cells (**Figures 6G, H** **)** to evaluate the effects on ovarian cancer cell proliferation. Intriguingly, we observed that WTAP knockdown almost completely abolished the proliferation inhibition levels resulting from METTL14 overexpression in SKOV-3 and A2780 cells (**Figure 6I** and **Supplementary Figure S4**), suggesting that there may be other mechanisms involved. These results suggest that METTL14 regulates TROAP expression *via* an m^6^A RNA methylation-dependent mechanism.

### METTL14 Suppresses Growth of Ovarian Cancer Xenografts *In Vivo*

**Figure 7:**
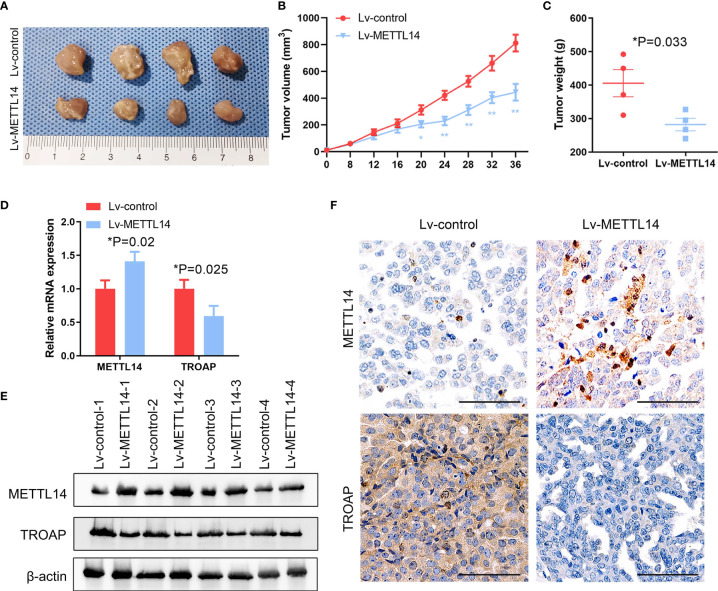
METTL14 inhibits ovarian cancer growth *in vivo*. **(A)** Control or METTL14–overexpressing lentivirus infected SKOV-3 cells were injected into nude mice, and tumor was evaluated after 36 days. **(B, C)** Tumor volume **(B)** and weight **(C)** at the end of the treatment period (**P < 0.01, *P < 0.05, *P = 0.033). **(D, E)** METTL14 and TROAP mRNA **(D)** and protein **(E)** expression levels assessed by real-time PCR and western blot analysis in tumors (*P = 0.02, *P = 0.025). **(F)** Xenograft tumor sections were subjected to IHC for METTL14 and TROAP. Magnification, 600 ×. Data are shown as mean ± SD from three independent experiments.

To confirm the function of METTL14 in ovarian cancer *in vivo*, we used the SKOV-3 cells with stable overexpression of METTL14 or control for establishing xenograft model. At 36 days after tumor cells inoculation, nude mice were sacrificed. The data indicated that METTL14 overexpression repressed the tumor volumes and weight (**Figures 7A–C**). The mRNA and protein expression of METTL14 were negatively correlated with TROAP in the tumor tissues dissected from the nude mice (**Figures 7D, E** **)**. In addition, histological analysis of xenograft tumors revealed that the tissues from METTL14-overexpression group mice had more METTL14 and less TROAP than from the control group (**Figure 7F**). Therefore, our results indicated that METTL14 inhibited the proliferation ability of human ovarian cancer cells *in vitro* and *in vivo*.

## Discussion

**Figure 8:**
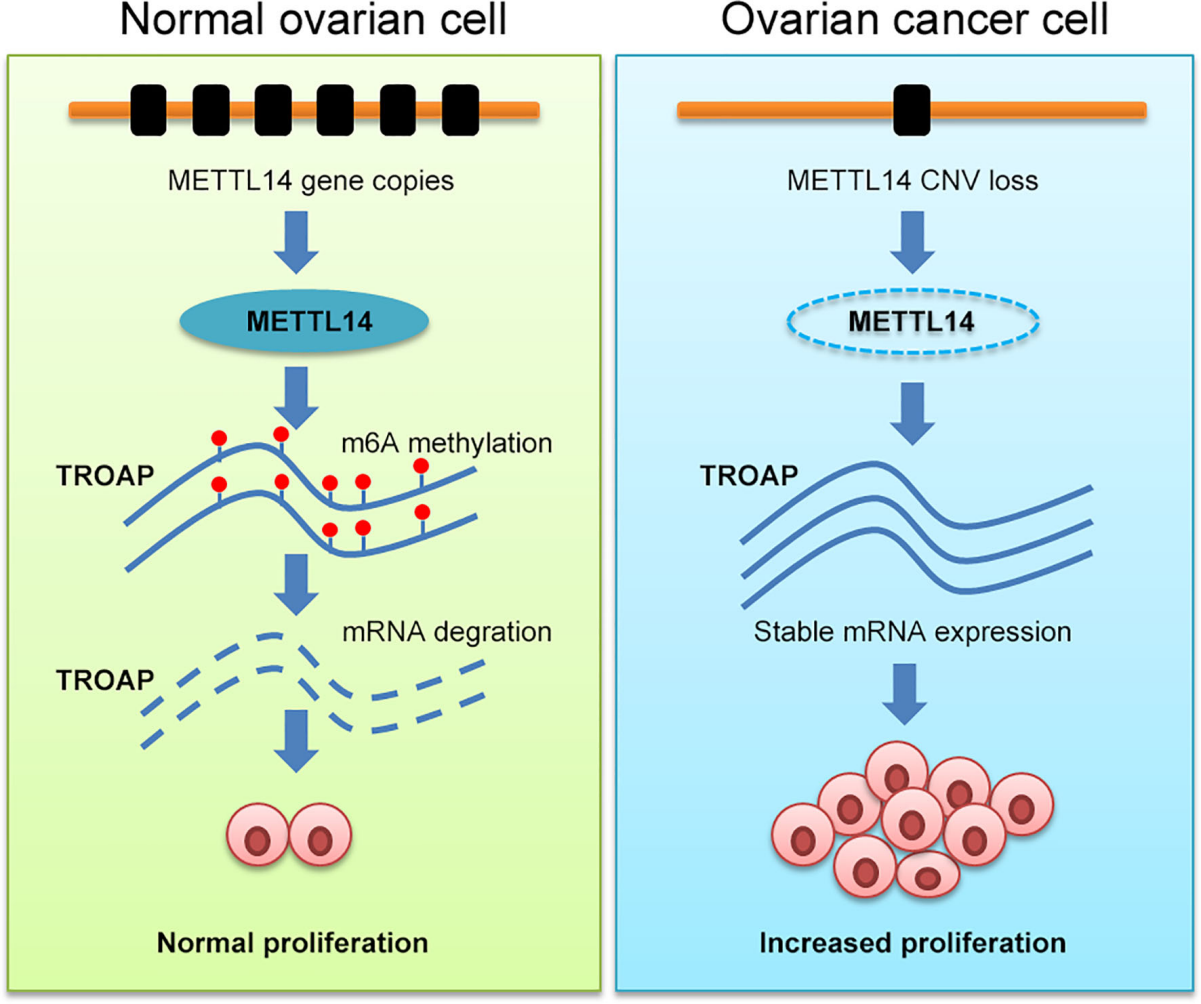
Schematic illustration of the regulatory role of METTL14 *via* its m^6^A RNA methylation function on TROAP expression and ovarian cancer cell proliferation.

In this study, we assessed and found considerable CNVs in the genes of the 10 selected regulators of m^6^A mRNA methylation in ovarian cancer tissues. Among these regulators, METTL14, which is an m^6^A mRNA methylation writer, also exhibited reduced expression levels and subsequent low levels of m^6^A methylation in ovarian cancer. Further investigation revealed that METTL14 reduced the stability of TROAP mRNA, which consequently arrested ovarian tumor cells at the G1 phase of the cell cycle and inhibited their proliferation. Our current data suggest a novel therapeutic target for the treatment of ovarian cancer (**Figure 8**).

Indeed, a previous study has reported that m^6^A modification improves the transcript stability of long non-coding RNA rhophillin Rho GTPase-binding protein 1-antisense RNA 1 (RHPN1-AS1) and reduces its degradation. Additionally, RHPN1-AS1 overexpression is associated with tumorigenesis and metastasis of ovarian cancer (11). However, the underlying mechanism of increased m^6^A RNA methylation remains unclear (12). Another recent study has shown that alterations in m^6^A modifications contribute to oncogenesis and at the same time represent an emerging class of therapeutic targets (3). Additionally, Li *et al.* have demonstrated that approximately 70% of all endometrial tumors exhibit a reduction in m^6^A methylation levels compared with that of normal endometrial tissues and that the downregulation or mutation of either METTL3 or METTL14 may be responsible for this reduction (6). In the current study, we assessed 10 regulators of m^6^A methylation for gene mutations and CNVs and found that reduced m^6^A modification levels were associated with the poor overall survival of ovarian cancer patients. Indeed, previous studies have shown that the induction of ALKBH5 expression occurs in ovarian cancer tissues and is associated with tumor cell proliferation and invasion *in vitro* (27). Elevated ALKBH5 expression has also been shown to contribute to the m^6^A modification of frizzled class receptor 10 (FZD10) mRNA and to the resistance of ovarian cancer cells to treatment with a poly-ADP ribose polymerase inhibitor, which may also be associated with altered FTO levels (28). Moreover, METTL3 expression is upregulated in ovarian cancer and is associated with advanced tumor grades and stages, lymph node and distant metastases, the International Federation of Gynecology and Obstetrics stage, and overall patient survival. Likewise, stable METTL3 overexpression significantly enhances tumor cell proliferation and invasion as well as tumor formation in nude mice (29). In addition, WTAP has been evaluated as a prognostic marker of high-grade serous ovarian cancer and is associated with tumor metastasis (30). The expression of YTHDF1, which is an m^6^A modification reader, is also associated with ovarian cancer progression (31). Similar to these findings, our current data showed that METTL14 had a significant reduction in CNVs in ovarian cancer tissues and that METTL14 overexpression inhibited ovarian cancer cell proliferation *in vitro*, further confirming the TCGA and GEO data. However, there are currently no previous reports on the role of METTL14 in ovarian cancer; thus, further studies are warranted. METTL14 is a critical part of the m^6^A methyltransferase complex and is responsible for binding to the RNA (32). Furthermore, it has been well documented that various methyltransferases play an important role in the growth and metastasis of multiple types of cancer, including renal clear cell carcinoma (8), hepatocellular carcinoma (5), and acute myeloid leukemia (33). Although increased METTL14 expression has been shown to suppress the progression of certain types of cancer, it leads to increased proliferation of acute myeloid cells.

TROAP (Trophinin-associated protein, also known as TASTIN) is found to participate in the proliferation, invasion, and migration of many cancers (34). Additionally, aberrant TROAP expression occurs in different human cancers (35). Recently, Jing *et al.* demonstrated that TROAP knockdown in gastric cancer cells results in reduced tumor cell growth, migration and invasion (36). Emerging evidence indicates that positive proliferation related gene, such as cyclin D1, survivin, and p-AKT, are positively regulated by TROAP (24–26). In the current study, we revealed TROAP as a downstream target of METTL14. We further demonstrated that METTL14 inhibited the expression of TROAP *via* the m^6^A RNA modification in ovarian cancer cells, which also inhibited cell proliferation by suppress cyclin D1, survivin, and p-AKT. Additionally, knockdown of TROAP reversed these inhibitory effects of METTL14 on tumor cell proliferation. However, we cannot exclude the possibility of other gene signaling pathways that may be involved in this regulatory mechanism. Therefore, additional investigations, including high-throughput analyses, are required to identify other possible genes that are targeted by METTL14.

In summary, the current study delineates that METTL14 negatively regulates TROAP expression in an m^6^A RNA methylation-dependent manner and that its expression is reduced in ovarian cancer and promotes cancer cell proliferation. These results suggest that the m^6^A modification plays a critical role in ovarian cancer growth and may be a novel therapeutic target that warrants further investigation.

## Data Availability Statement

The datasets presented in this study can be found in online repositories. The names of the repository/repositories and accession number(s) can be found in the article/**Supplementary Material**.

## Ethics Statement

The studies involving human participants were reviewed and approved by Ethics Committee of the First Affiliated Hospital of Fourth Military Medical University. The patients/participants provided their written informed consent to participate in this study The animal study was reviewed and approved by Animal Care Committee of the Fourth Military Medical University.

## Author Contributions

WL and WB conceived and designed the study. YL, HP, PJ, JZ, and XF performed the *in vitro* analysis. YZ, CP, and JR analyzed the data and prepared the figures. HZ, WB, and WL wrote the manuscript. All authors contributed to the article and approved the submitted version.

## Funding

This study was supported by the National Natural Science Foundation of China (81702554 to YL, 81802661 to WB).

## Conflict of Interest

The authors declare that the research was conducted in the absence of any commercial or financial relationships that could be construed as a potential conflict of interest.

## Publisher’s Note

All claims expressed in this article are solely those of the authors and do not necessarily represent those of their affiliated organizations, or those of the publisher, the editors and the reviewers. Any product that may be evaluated in this article, or claim that may be made by its manufacturer, is not guaranteed or endorsed by the publisher.
