# Supplementary material for: Downregulation of Methyltransferase-Like 14 Promotes Ovarian Cancer Cell Proliferation Through Stabilizing TROAP mRNA
Source: Front Oncol. 2022 Feb 18;12:824258. doi: 10.3389/fonc.2022.824258 (PMC8894193; doi:10.3389/fonc.2022.824258)
Supplement: Supplementary file 1 [file DataSheet_1.docx]

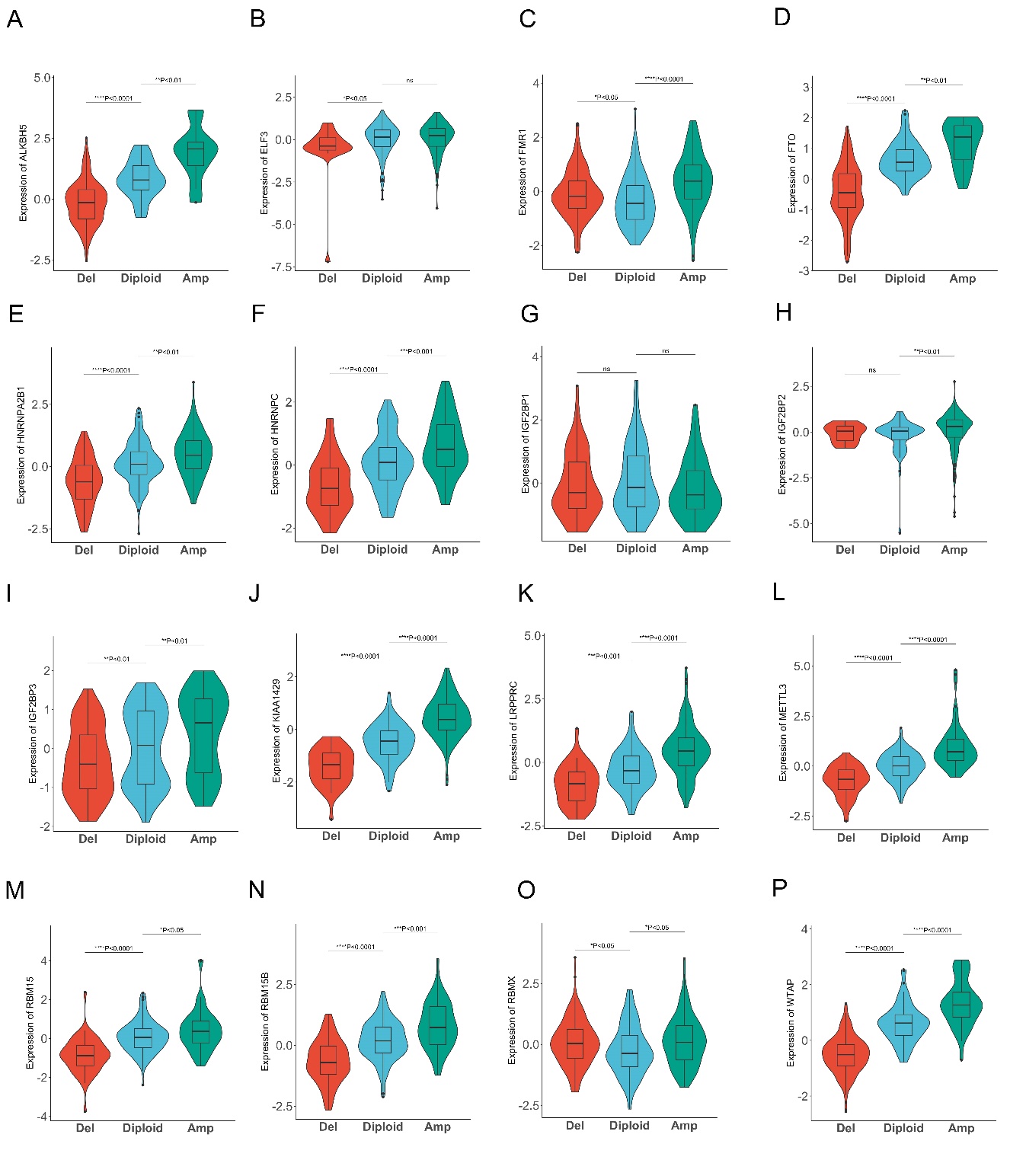


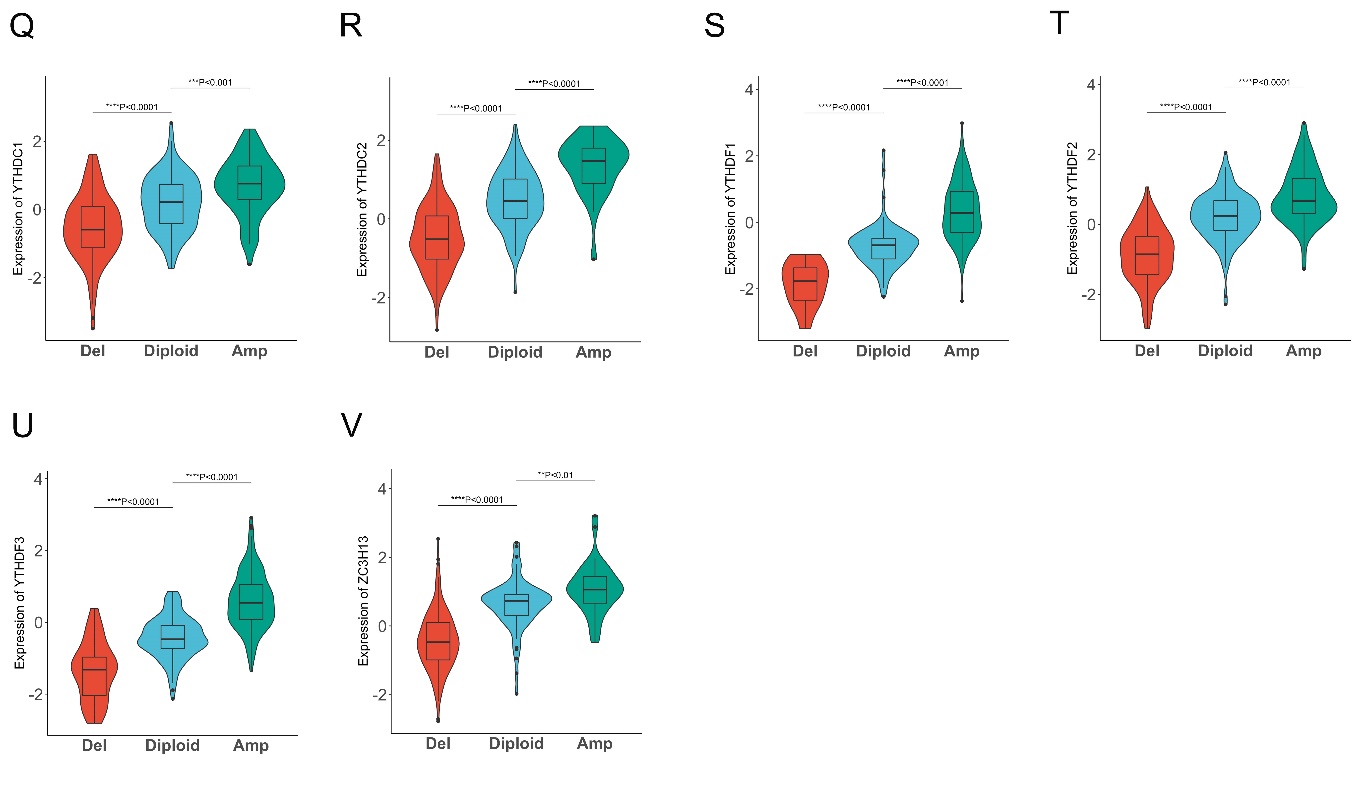


**FIGURE S1 |** Computational analysis of oncogenomic data of the 22 m^6^A RNA methylation regulators besides METTL14. This dataset was used to analyze the correlation between the copy number category (deletion, diploid and amplification) and the expression level (*P < 0.05, **P < 0.01, ***P < 0.001, ****P < 0.0001). Graphical data represented as the mean ± SD.


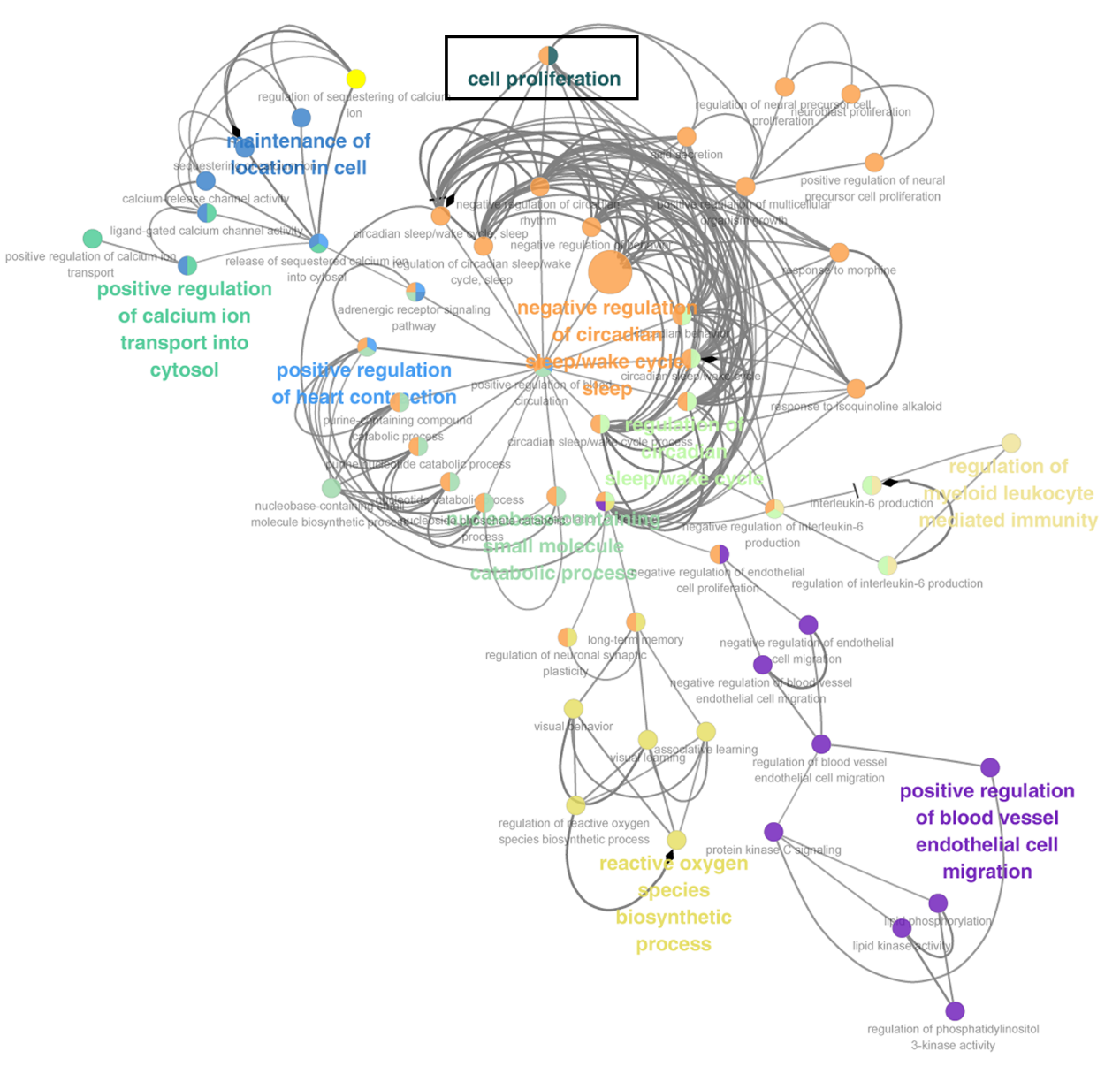


**FIGURE S2 |** Pathway enrichment analysis using the ClueGO and CluePedia plugins found in the Cytoscape software. Cluster analysis showing the most inversely correlated genes with METTL14 using the GEO data (GSE14407).


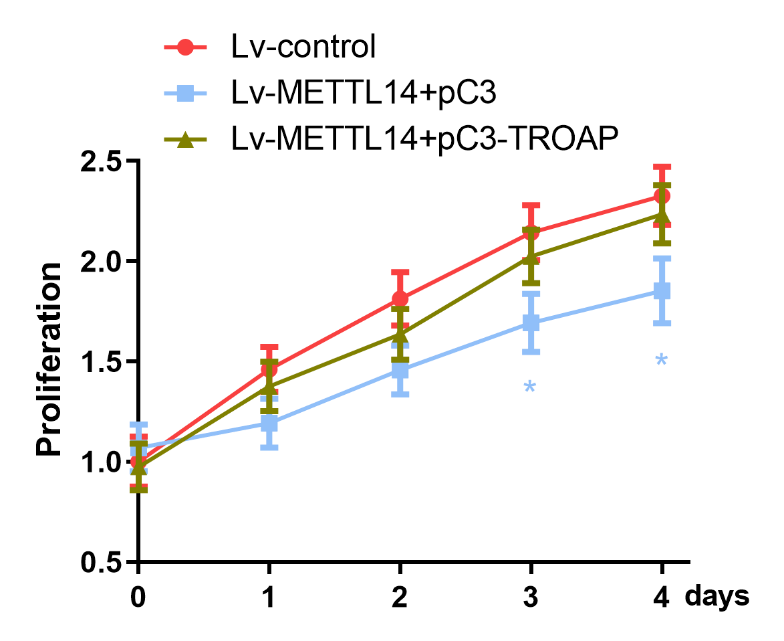


**FIGURE S3 |** MTS assay of METTL14-overexpressing A2780 cells transduced with pC3 or pC3-TROAP. Values at the indicated timepoints are provided as the mean absorbance with a standard deviation of 6 wells (*P<0.05). Graphical data represented as the mean ± SD.


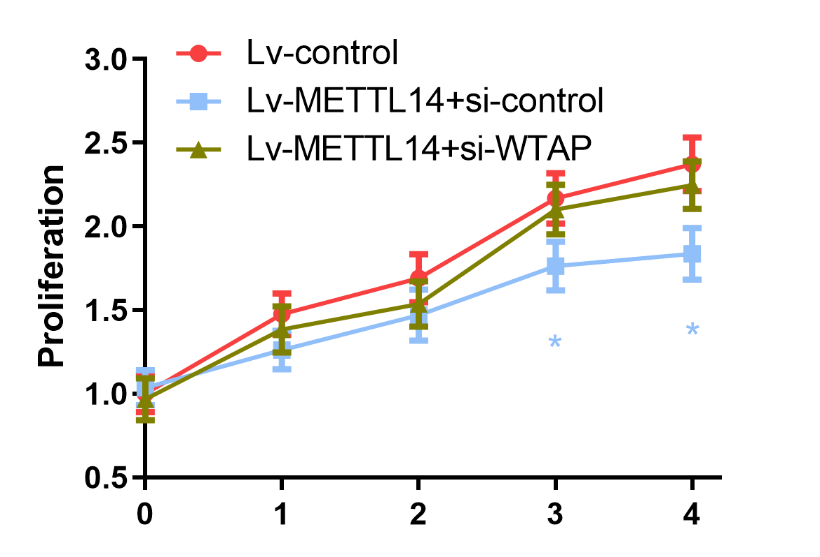


**FIGURE S4 |** Cell proliferation analysis of stable METTL14-overexpressing A2780 cells transfected with si-control or si-WTAP (*P<0.05). Graphical data represented as the mean ± SD.
